# Experience-based VAS values for EQ-5D-3L health states in a national general population health survey in China

**DOI:** 10.1007/s11136-014-0793-6

**Published:** 2014-09-24

**Authors:** Sun Sun, Jiaying Chen, Paul Kind, Ling Xu, Yaoguang Zhang, Kristina Burström

**Affiliations:** 1 Department of Learning, Informatics, Management and Ethics, Medical Management Centre, Karolinska Institutet, Tomtebodavägen 18A, 171 77 Stockholm, Sweden; 2 Department of Public Health Sciences, Equity and Health Policy Research Group, Karolinska Institutet, Stockholm, Sweden; 3 Stockholm County Council, Health Care Services, Stockholm, Sweden; 4 School of Health Policy and Management, Nanjing Medical University, 140 Hanzhong Rd, Nanjing, 210029 China; 5 Leeds Institute of Health Sciences, University of Leeds, Leeds, UK; 6 Centre for Health Statistics and Information, Ministry of Health, Beijing, China

**Keywords:** China, EQ-5D, Experience-based values, General population, Health status, Visual analogue scale

## Abstract

**Purpose:**

To investigate the feasibility of deriving experience-based visual analogue scale (VAS) values for EQ-5D-3L health states using national general population health survey data in China.

**Methods:**

The EQ-5D-3L was included in the National Health Services Survey (*n* = 120,709, aged 15–103 years) to measure health-related quality of life. The respondents reported their current health status on a VAS and completed the EQ-5D-3L questionnaire, enabling modelling of the association between the experience-based VAS values and self-reported problems on EQ-5D dimensions and severity levels.

**Results:**

VAS values were generally negatively associated with problems reported on the EQ-5D dimensions, and the anxiety/depression dimension had the greatest impact on VAS values. A previously obtained value for dead allowed the values for all 243 EQ-5D-3L health states to be transformed to the 0–1 scale (0 = dead, 1 = full health).

**Conclusions:**

This study presents the feasibility of deriving an experience-based VAS values for EQ-5D-3L health states in China. The analysis of these VAS data raises more fundamental issues concerning the universal nature of the classification system and the extent to which Chinese respondents utilise the same concepts of health as defined by this classification system.

**Electronic supplementary material:**

The online version of this article (doi:10.1007/s11136-014-0793-6) contains supplementary material, which is available to authorized users.

## Background

EQ-5D is a widely used generic health-related quality of life (HRQoL) instrument [1], with applications in clinical studies, economic evaluation of health care [2] and in population health surveys [3]. It is recommended by the UK National Health Service (NHS) as a health outcomes measure for use by clinicians and managers [4]. In China, there is an increasing interest in applying EQ-5D, both amongst patients [5, 6] and the general population [7–10]. EQ-5D-3L (with five dimensions and three severity levels) defines a classification of 243 health states and was included in the National Health Services Survey (NHSS) 2008 in China, and population norms have been established by age, sex, socioeconomic status [8] and geographic area [9].

EQ-5D-3L health states represent a nominal level of measurement since they cannot be ordered and have no intrinsic quantitative score. In order to convert such a classification into a cardinal scale with true arithmetic properties, it is necessary to devise a system whereby individual health states can be assigned an index value. Methods for deriving scores for use in economic evaluation must take into account several important methodological considerations, in particular, which valuation method should be used and whose values should be applied. Many methods have been used to obtain health state values including: standard gamble (SG), time trade-off (TTO) and rating scale (RS) [2]. Both TTO and RS (visual analogue scale (VAS)) have been used for obtaining EQ-5D value sets [11, 12], and recently, the discrete choice method was tested [13]. However, none of these methods is recognised as being the standard measure for valuing health in economic evaluations [2, 14].

Similarly, there are differences of opinion as to whose values should be used [14–17]: experience-based values are based on assessments made by individuals who are actually in the health state; hypothetical values are based on assessments of health state descriptions. Experience-based values for EQ-5D-3L health states have been investigated, both for TTO [16, 18, 19] and VAS [16, 18, 20–22]. Previous studies have shown that the experience-based values tend to be higher than hypothetical values [15, 16, 22–28], and the anxiety/depression dimension seems to be more important when values are experience-based [16, 22–25]. For EQ-5D valuation studies based on hypothetical values, in general, the VAS values are higher than those using TTO values [12, 29]. As far as is known, only two studies [16, 18] have reported both TTO and VAS values from the same respondents using experience-based values.

The aim of the present study is to investigate the feasibility of deriving experience-based VAS values for EQ-5D-3L health states using national general population health survey data in China.

## Materials and methods

### Material/study population

Data are obtained from the National Health Services Survey 2008 (NHSS 2008), which is organised by the Ministry of Health (MoH). A multi-stage stratified cluster random sampling method was used, in total, 177,051 respondents were face-to-face interviewed. Of these, about 18 % aged below 15 years were excluded. Respondents needing assistance in answering questions were excluded (13 %) as were those who had missing answers on age, sex, in at least one of the EQ-5D dimensions and on VAS. These accounted for a further 2 %. For 6 respondents with a profile of 11111 and VAS higher than 100, their VAS value were imputed as 100. After applying these criteria, 120,709 respondents were available for further study. The NHSS sampling design was examined by the MoH for all waves of the surveys, and the representativeness of the sample was considered good, i.e., proportions of the population from different regions, age, sex and socio-economic structures are representative of the Chinese population and are similar to the census data, except for the unemployment rate, which might be due to different ways of defining unemployment [30].

Details regarding questionnaire, sampling method, interview procedure can be found elsewhere [8, 9]. The value for dead was obtained from the Household Health Survey 2010 (*n* = 8,031), which used a similar protocol as the NHSS 2008.

The EQ-5D-3L instrument classifies respondents’ health status in five dimensions (mobility, self-care, usual activities, pain/discomfort and anxiety/depression), with three severity levels (no problems, some problems and severe problems), which in total defines 243 health states [1]. The VAS consisted of a horizontal 11 cm line where every tenth was marked and labelled 0, 10, 20, …, 100, with anchor points 0 (worst health state) and 100 (best health state). The question was framed: ‘On the scale please point out which point best represents your own health state today’. Respondents were asked to record their value for the state ‘dead’ using the same VAS. The question was framed: ‘This scale is the same as the one you saw before. On this scale, where would you score dead?’ The scale was harmonised to fit in the NHSS questionnaire and hence differs from the EQ VAS.

Ethical permissions have been granted by the Regional Ethics Committee, Stockholm, Sweden for the studies (Dnr: 2009/1892-31/5, for NHSS 2008; Dnr: 2011/581- 31/5, for HHS 2010).

### Data analyses

All statistical analyses were performed using SAS version 9.2 [31], using a 5 % significance level. Ordinary least square (OLS) was used for all regression analysis. Definition of variables and models are presented in Table 1. A structured approach to data analysis was taken in which a basic main effects model was specified using two dummy variables for each of the five dimensions. The performance of alternative models was examined in which interaction terms were included with a view to improving model performance. Interaction terms were included as follows: if any dimension is on level 2 or 3 (N2 and N3, respectively), number of dimensions at level 2 or 3 beyond the first one and the square term of it. However, only N3 leads to consistent results, and therefore, we only present models with N3 term.

**Table 1:** Definition of variables and models

Variable	Definition
MO2	1 If mobility is level 2; 0 otherwise
MO3	1 If mobility is level 3; 0 otherwise
SC2	1 If self-care is level 2; 0 otherwise
SC3	1 If self-care is level 3; 0 otherwise
SC3*	1 If self-care is level 3; 0 otherwise (merged levels 1 and 2 in the reference group)
SC23	1 If self-care is level 2 or 3; 0 otherwise
UA2	1 If usual activities is level 2; 0 otherwise
UA3	1 If usual activities is level 3; 0 otherwise
PD2	1 If pain/discomfort is level 2; 0 otherwise
PD3	1 If pain/discomfort is level 3; 0 otherwise
AD2	1 If anxiety/depression is level 2; 0 otherwise
AD3	1 If anxiety/depression is level 3; 0 otherwise
N3	1 If any dimension is level 3; 0 otherwise

Models	*f*(*x*)
*Models based on individual data*	
Model 1	*f* (mo2 mo3 sc2 sc3 ua2 ua3 pd2 pd3 ad2 ad3)
Model 2	*f* (mo2 mo3 sc2 sc3 ua2 ua3 pd2 pd3 ad2 ad3 N3)
Model 3	*f* (mo2 mo3 sc3* ua2 ua3 pd2 pd3 ad2 ad3)
Model 4	*f* (mo2 mo3 sc3* ua2 ua3 pd2 pd3 ad2 ad3 N3)
Model 5	*f* (mo2 mo3 sc23 ua2 ua3 pd2 pd3 ad2 ad3)
Model 6	*f* (mo2 mo3 sc23 ua2 ua3 pd2 pd3 ad2 ad3 N3)
*Models based on aggregated data*	
Model 1M1–Model 1M3	*f* (mo2 mo3 sc2 sc3 ua2 ua3 pd2 pd3 ad2 ad3)

A primary requirement for any estimation model is that coefficients are monotonically consistent within dimensions so that value loss increases as the level of problem becomes greater. Firstly, we tested the models with the ten dummy variables (Model 1). However, the coefficient for moderate problems on self-care dimension (SC2) was positive; therefore, we tested N3 term, but SC2 was still positive. Two further sets of models were tested. In Models 3 and 4, SC2 was excluded, and thus for self-care dimension, the levels 1 and 2 were merged into one category in the reference group and the coefficient for self-care level 3 was then represented by SC3*. In Models 5 and 6, for self-care dimension, the levels 2 and 3 were merged into one category, by including a new dummy variable SC23. F-tests were used to make comparisons between the models with and without the N3 term.

For models based on individual-level data, raw VAS value was used as the dependant variable in the OLS models. Due to the skewed distribution of data, we have tested OLS models with log-transformed VAS [32]. Furthermore, we also performed Poisson, negative binomial [21, 33], Tobit [34] and quantile models [35]. However, compared with the OLS models, these did not provide better results, in terms of monotonicity and goodness of fit; therefore, we only present OLS models with raw VAS value.

The survey dataset contains multiple ratings from separate individuals who classify themselves in the same EQ-5D health state. For these health states, it is possible to compute a mean rating which represents the average VAS value associated with that specific health state. Step-wise sensitive analyses were taken, to investigate how many observations were required in order to generate a ‘safe’ mean for each EQ-5D health state (results can be provided on request). In this study, EQ-5D health states with 20 or more observations were considered reasonable. Aggregate-level analyses were carried out using models in which mean VAS value for these health states were taken as the dependent variable and the ten main effect dummy variables as the independent variables.

Selection of the final models is based on the following criteria [36]: the model should be simple (parsimony), should provide consistent results with an acceptable goodness of fit and should be transparent so as to be able to be understood by non-experts. Spearman rank correlation coefficients (SCC) and mean absolute difference (MAD) were used to examine the goodness of fit of the models. Higher SCC and lower MAD indicates better model fitting.

We employed a split sample test in order to estimate the robustness of the final model. Furthermore, we explored the effect of socio-demographic factors on health state valuation. Details regarding the above analyses can be found in online resource (QURE-S-14-00050_ESM.pdf).

## Results

Characteristics of the sample, percentage of problems reported on each EQ-5D dimension and mean VAS score are presented in Table 2.

**Table 2:** Characteristics of the respondents

	15–103 years (*n* = 120,709)	
	%	*n*
*Sex*		
Men	48.2	58,169
Women	51.8	62,540
*Age group (years)*		
15–24	11.3	13,635
25–34	13.7	16,510
35–44	23.3	28,088
45–54	21.3	25,695
55–64	16.2	19,557
65–74	9.5	11,491
75–103	4.8	5,733
*Region*		
Urban	27.7	33,447
Rural	72.3	87,262
*Area*		
Eastern	35.1	42,305
Middle	27.5	33,175
Western	37.5	45,229
*Marital status*		
Single	11.9	14,406
Married	79.2	95,649
Divorced	1.4	1,744
Widowed	7.1	8,605
Other	0.2	234
Missing	0.1	71
*Educational level*		
Below primary school	15.6	18,841
Primary school	27.9	33,630
Junior middle school	35.7	43,042
Senior middle school	14.9	17,941
College and above	5.9	7,160
Missing	0.1	95
*Income groups*		
First group (low)	22.8	27,560
Second group	21.6	26,037
Third group	18.9	22,791
Fourth group	17.7	21,417
Fifth group (high)	19.0	22,904
*Occupational status*		
Employed	70.6	85,161
Retired	10.2	12,313
Student	4.4	5,322
Unemployed	14.6	17,627
Missing	0.2	286
*EQ-5D dimension*		
*Mobility*		
Moderate problems (level 2)	4.8	5,760
Severe problems (level 3)	0.4	447
*Self-care*		
Moderate problems (level 2)	2.8	3,413
Severe problems (level 3)	0.4	522
*Usual activities*		
Moderate problems (level 2)	4.0	4,850
Severe problems (level 3)	0.8	978
*Pain/discomfort*		
Moderate problems	8.8	10,661
Severe problems	0.4	500
*Anxiety/depression*		
Moderate problems (level 2)	6.0	7,287
Severe problems (level 3)	0.4	467

	Mean	SD
VAS score	80.1	14.1

The observed EQ-5D health states are presented in online resource Supplementary Table S1. In total, 167 out of the 243 possible EQ-5D health states were observed and 51 health states had 20 or more observations. The most frequently occurred health state was 11111 (87 % of the population), followed by 11121 and 11112. The mean VAS value for 11111 was 82.6, which was 17 points below the upper boundary of best health state. The mean VAS value for 33333 was 34, which was 34 points above the lower bound of worst health state.

Table 3 shows the coefficients produced by OLS based on individual-level data. Models 1 and 2 included all the ten dummy variables; the coefficients were monotonic except for SC2. In Models 3 and 4, all coefficients were monotonic. In Models 5 and 6, coefficient for self-care dimension and N3 were positive. In Model 4, the N3 terms were negative and significant. However, the F-test did not suggest that the Model 4 was significantly better than Model 3.

**Table 3:** Regression analysis on VAS values and EQ-5D dimensions, individual-level data

	Model 1		Model 2		Model 3		Model 4		Model 5		Model 6	
	Estimate	*p* value	Estimate	*p* value	Estimate	*p* value	Estimate	*p* value	Estimate	*p* value	Estimate	*p* value
Intercept	82.39	<0.0001	82.39	<0.0001	82.39	<0.0001	82.39	<0.0001	82.39	<0.0001	82.39	<0.0001
Mobility												
Level 2	−6.55	<0.0001	−6.53	<0.0001	−6.39	<0.0001	−6.35	<0.0001	−6.52	<0.0001	−6.49	<0.0001
Level 3	−8.36	<0.0001	−8.10	<0.0001	−8.13	<0.0001	−7.86	<0.0001	−9.48	<0.0001	−9.23	<0.0001
Self-care												
Level 2	0.74	0.0212	0.80	0.0126	–	–	–	–	–	–	–	–
Level 3	−2.69	0.0008	−2.66	0.0009	–	–	–	–	–	–	–	–
Level 3^a^	–	–	–	–	−3.21	<0.0001	−3.22	<0.0001	–	–	–	–
Level 2 and 3	–	–	–	–	–	–	–	–	0.57	0.76	0.63	0.0500
Usual activities												
Level 2	−6.18	<0.0001	−6.14	<0.0001	−5.89	<0.0001	−5.83	<0.0001	−6.11	<0.0001	−6.07	<0.0001
Level 3	−11.53	<0.0001	−8.93	<0.0001	−11.21	<0.0001	−8.64	<0.0001	−12.49	<0.0001	−9.91	<0.0001
Pain/discomfort												
Level 2	−11.07	<0.0001	−11.04	<0.0001	−11.07	<0.0001	−11.04	<0.0001	−11.06	<0.0001	−11.04	<0.0001
Level 3	−13.98	<0.0001	−12.76	<0.0001	−13.98	<0.0001	−12.78	<0.0001	−14.34	<0.0001	−13.13	<0.0001
Anxiety/depression												
Level 2	−8.44	<0.0001	−8.39	<0.0001	−8.41	<0.0001	−8.37	<0.0001	−8.43	<0.0001	−8.39	<0.0001
Level 3	−16.68	<0.0001	−14.93	<0.0001	−16.63	<0.0001	−14.90	<0.0001	−16.79	<0.0001	−15.04	<0.0001
N3	–	–	−3.45	<0.0001	–	–	−3.39	<0.0001	–	–	3.43	0.0001
*Observations*	120,709		120,709		120,709		120,709		120,709		120,709	
*Adj R* ^*2*^	0.2405		0.2406		0.2404		0.2406		0.2404		0.2406	

*F*-tests have been performed between models with and without N3 term, no result is significant at *p* = 0.05 level

^a^Reference group: self-care on level 1 or 2

Table 4 shows the coefficients produced by OLS based on aggregated data. The coefficients are monotonic for all dimensions in all models. For level 3, anxiety/depression had the greatest coefficient, followed by pain/discomfort and usual activities. For level 2, pain/discomfort had largest coefficient, followed by mobility and anxiety/depression. Overall, by excluding health states with fewer observations in the analyses, the adjusted R^2^ improved from Model 1M1 (0.91) to Model 1M3 (0.93).

**Table 4:** Regression analysis on VAS values, EQ-5D dimensions, aggregated data

	Model 1M1^a^		Model 1M2^b^		Model 1M3^c^	
	Estimate	*p* value	Estimate	*p* value	Estimate	*p* value
Intercept	74.12	<0.0001	74.30	<0.0001	75.03	<0.0001
Mobility						
Level 2	−4.49	0.0002	−4.53	0.0003	−4.92	<0.0001
Level 3	−5.88	0.0082	−4.77	0.0355	−4.63	0.0301
Self-care						
Level 2	−0.78	0.4754	−0.60	0.6025	−0.85	0.3903
Level 3	−6.99	0.0028	−5.54	0.0264	−5.08	0.0397
Usual activities						
Level 2	−4.85	0.0001	−5.11	0.0001	−5.50	<0.0001
Level 3	−9.79	<0.0001	−10.58	<0.0001	−11.04	<0.0001
Pain/discomfort						
Level 2	−6.36	<0.0001	−5.96	<0.0001	−5.57	<0.0001
Level 3	−9.85	<0.0001	−10.23	<0.0001	−10.76	<0.0001
Anxiety/depression						
Level 2	−5.13	<0.0001	−5.48	<0.0001	−5.65	<0.0001
Level 3	−12.39	<0.0001	−12.86	<0.0001	−15.27	<0.0001
Adjusted R^2^	0.9076		0.9136		0.9308	

^a^Health states with less than 20 observations are excluded (number of health states = 51)

^b^Health states with less than 25 observations are excluded (number of health states = 47)

^c^Health states with less than 30 observations are excluded (number of health states = 43)

**Fig. 1:**
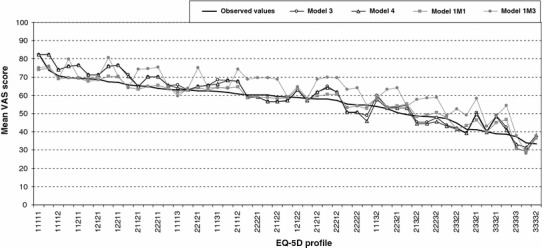
Observed values compared with predicted values from different OLS models for the most frequently occurred health states

The estimated values predicted by different models were compared with the observed values (Fig. 1), and goodness-of-fit statistics were reported (Table 5). For health states with 20 or more observations, for individual-level data, Models 3 and 4 performed the best; for aggregated data, it was Model 1M1.

**Table 5:** Spearman rank correlation coefficients (SCC) and mean absolute difference (MAD)

Observations in each health state	Number of health states	Individual-level data							
		Model 1		Model 2		Model 3		Model 4	
		Corr	MAD	Corr	MAD	Corr	MAD	Corr	MAD
*n* ≥ 1	167	0.686	7.55	0.676	7.51	0.687	7.53	0.677	7.48
*n* ≥ 20	51	0.945	4.14	0.946	3.97	0.945	4.10	0.947	3.92
*n* ≥ 25	47	0.950	4.07	0.951	3.91	0.951	4.03	0.952	3.87
*n* ≥ 30	43	0.952	3.90	0.951	3.84	0.953	3.85	0.952	3.78

Observations in each health state	Number of health states	Aggregated data					
		Model 1M1		Model 1M2		Model 1M3	
		Corr	MAD	Corr	MAD	Corr	MAD
*n* ≥ 1	167	0.668	7.02	0.643	8.83	0.655	8.88
*n* ≥ 20	51	0.960	2.30	0.871	6.13	0.864	6.72
*n* ≥ 25	47	0.962	2.23	0.871	6.13	0.865	6.70
*n* ≥ 30	43	0.964	1.99	0.861	5.95	0.855	6.63

The parsimony, monotonicity criteria and F-test (Table 3), and goodness-of-fit analyses (Table 5; Fig. 1) suggested that for the individual-level data, Model 3 was the best-fitting model; for the aggregated data, it was Model 1M1. For Model 3, the intercept was 82.4, corresponded to the observed mean value for health state 11111 (82.6). Coefficients for level 3 and level 2 were compared in absolute terms. For level 3, the greatest coefficient was seen for anxiety/depression (16.6), followed by pain/discomfort (14.0) and usual activities (11.2). For level 2, the greatest coefficient was seen for pain/discomfort (11.1), followed by anxiety/depression (8.4) and mobility (6.5). For Model 1M1, the intercept was 74.1, which was about 9 points lower than the observed value for 11111. For level 3, the greatest coefficient was seen for anxiety/depression (12.4), followed by pain/discomfort (9.9) and usual activities (9.8). For level 2, the greatest coefficient was seen for pain/discomfort (6.4), followed by anxiety/depression (5.1) and mobility (4.5).

Based on Model 3 (individual-level data) and Model 1M1 (aggregated data), VAS values for all the 243 EQ-5D-3L health states can be calculated. The VAS had the endpoints worst and best health state, which did not allow for anchoring between 0 (dead) and 1 (full health). For using VAS values in quality-adjusted life year (QALY) calculations, rescaling by the value for dead is needed. In this present study, the value for dead was obtained from the Household Health Survey 2010. The mean value for dead was 4.5. So as to rescale the estimated VAS values on a 0–1 metric, the formula (VAS_Estimated _− dead_mean_)/(VAS_11111 _− dead_mean_) [11] was used. The estimated and rescaled VAS values for 243 EQ-5D health states are presented in Table S2 in online resource.

## Discussion

Our study reports on the estimation of experience-based VAS values for EQ-5D-3L health states, using data from a large national cross-sectional population-based survey conducted in China. In the NHSS 2008, individuals reported their current health status using the EQ-5D descriptive system and valued their health using VAS. Appropriate sampling methods were used to recruit a national representative sample, which is the strength of our study. Furthermore, by utilising a previously obtained value for dead, we are able to transform values for all the 243 EQ-5D health states to a 0–1 scale (0 = dead; 1 = full health).

In China, 167 out of 243 EQ-5D health states were observed; this number is higher than that recorded in Sweden (148) [16] and UK (139) [20], where experience-based VAS values were also used to derive values for EQ-5D-3L health states. That more health states were observed in this study than in Sweden and UK, might be due to the larger sample size of the Chinese survey. In all countries, the most frequently occurring health state was 11111, followed by 11121. For China and UK, 11112 was the third; for Sweden, it was 11122. Nearly, 87 % of the respondents reported 11111 in China, higher than UK (45 %), Sweden (42 %) and Germany (66 %) [21]. The rate of respondents reporting no problems on EQ-5D dimensions in this present survey is roughly double the rate observed elsewhere and warrants further investigation.

Anxiety/depression has the greatest impact on overall HRQoL, as suggested in other studies in which experience-based values were used [16, 21, 22]. The difference between hypothetical values and experience-based values might be due to adaptation, contrast effects and shifting comparisons [19]. In the hypothetical valuation, the respondents might over-estimate loss in health as they underestimate the adaptation, and focus on transitory change from one health state to another [24]. Our study is in line with previous studies [15, 16, 18, 22–28] and shows that the experience-based values tend to be higher than hypothetical values. The use of experience-based values in an intervention may seemingly lead to a smaller gain comparing with if values were based on hypothetical health states. If this is an underestimation of the gain depends on whose preferences are considered most suitable.

Several estimation models produced evidence of non-monotonicity, which was encountered by other studies as well [16, 20, 37–39]. By merging self-care levels 1 and 2, the results are more logical; however, the index value is insensitive to the difference between levels 1 and 2 on self-care dimension. The reason for the observed non-monotonicity probably stems from construct–irrelevant variance or construct underrepresentation. We observed that a few respondents (1 %) reported problems on EQ-5D dimensions, yet had a VAS value at 100 (best heath); some respondents reported no problem, but reported a very low VAS value. This might due to misunderstanding, measurement noise, or that respondents actually valued own health state like that. As it is difficult to define what could be the most reasonable range of VAS value for a certain health state, we included all the answers in the analysis. Another issue is regarding the inconsistent pairs in the observed values, for example, 12222 is logically worse than 11222, but we observed a higher value for 12222 than 11222. We have identified all these kinds of logical inconsistent pairs, most of them were due to the small number of observation for that health state. That is also why we only report SCC and MAD for the health states with 20 or more observations. In our data, most inconsistency pairs came from the self-care dimension. The non-monotonicity for the self-care dimension might be due to the above reason, but also the skewness of the data, multi-collinearity and heteroscedasticity might contribute to that [20, 37].

The valuations for respondent’s own health seen in the data collected in this study suggest a truncated use of the VAS rating scale, with gaps evident at both the higher and lower range. Respondents who self-classify as being in the 11111 health state report a mean VAS rating that is some 17 points less than the defined value assigned to best imaginable health. Similarly, the high value for dead creates a 34 point gap between dead and worst imaginable health. This discontinuity in values might be a result of the valuation method itself, or a by-product of the descriptive classification. Whatever is the cause, it suggests that there are other mechanisms at work here that are yet poorly understood.

Taken together, these results suggest that there might be health domains additional to those specified as EQ-5D dimensions [20, 40], so that respondents might not consider that 11111 is in fact the best (or even best imaginable) health state [41]. The high proportion of respondents reporting 11111 in China might be attributable to many causes linked to the EQ-5D descriptive classification, for example at a purely technical level, the process of translation may have introduced incorrect meaning to the health problem descriptions. However, this seems unlikely although the hugely skewed distribution of responses would be consistent with the presence of an intrinsic design flaw.

Given the magnitude of the phenomenon, it may be that the model of health that provides the conceptual foundation of EQ-5D is simply not recognised by respondents with the Chinese or other East Asian culture background in the same way that it is by (say) respondents with purely Western European or North American culture background [3, 7, 40, 42, 43]. Culture can impact respondents answers from several perspectives [44]. For example, the numbers might be used differently cross different cultures, e.g., whether or not 100 on a VAS scale means the same thing across different cultures; or some items might function differently in different cultures, e.g., comparing with the English, Spanish and French respondents, the Chinese respondents consider the word ‘moderate problems’ representing more severe degree than other countries [45]. Whilst the EQ-5D dimensions themselves may appear to be relevant in describing health, the concept of varying degrees of problems within each dimension might not be recognised in the same way. Additional exploration of the concept of ‘health’ in China also seems necessary.

Both TTO and VAS have been adopted as valuation methods for eliciting values for the EQ-5D health states [11, 12]. VAS value sets are available for Belgium [46], Malaysia [47] and Europe [32]. VAS might be considered to be inferior to TTO, as it is not a choice based measure [2]; however, other views can be seen as well [48, 49]. If QALY is applied in non-economic evaluation, such as monitoring health status change of a population, then there is no requirement that the quality–adjustment factor must be a utility measure [20]. As stated by the EuroQoL Group that ‘the theoretical and empirical case for favouring one method of health state valuation over another is far from clear cut. In practice, there are currently no EQ-5D value set generated from SG methods, so for users the choice is between TTO and VAS’ [11]. The focus of the present study is to raise methodological issues, and further investigations are needed; hence, the rescaled values from this study should not be treated as EQ-5D tariff.

The presentation of results based both on the analysis of individual-level data and the aggregated data for observed health states raises important questions for analysts and decision-makers. Theoretically, the analyses based on individual-level data are expected to produce better results as they take each individual’s information into consideration. However, this approach relies upon there being reasonable parity amongst the health states under consideration. In valuation studies based on hypothetical health states, considerable care will be taken in selecting the states to ensure roughly comparable coverage per dimension/level. In a study based on experience-based health states, however, such control of design is infeasible. The fact that the vast majority of respondents report having no problem on any of the EQ-5D dimensions resulted in a skewed distribution of data, which in itself presents problems, especially with low frequencies of reported problems evident for some dimensions such as self-care and mobility. This has implications for the design of any study that seeks to establish experience-based values. Estimating a model based on aggregated data has the twin advantage of smoothing the variability of VAS values present for each state whilst at the same time reducing the potential swamping of minority health states by the overwhelming presence of the 11111 health state. Of course, there are drawbacks to this approach, notably in estimating mean values for health states with relatively few observations. This naturally raises the question as to how many observations are required in order to generate a ‘safe’ mean. We have tried some sensitive analysis in this study, however, to which extend it fits the requirement of power calculations based on statistical theory and how important that is, is for further research. But as long as we are cautious in our interpretation of results, we can still make progress with our understanding. In this study, for individual-level data, Model 3 performed best. However, by merging self-care levels 1 and 2, the index value is insensitive to the difference between levels 1 and 2 on self-care dimension, and for example, 11111 and 12111 would have the same index value [21]. For aggregated data, Model 1M1 performs best, however, the big gap between the estimated value and the observed value for the health state 11111 is problematic, which needs further investigation. The purpose of the present study was to help us gain a better understanding of the methodological issues that confront us in developing a mechanism for valuing EQ-5D health states using experience-based VAS values. Hence, it would be premature to suggest at this early stage that whether models based on individual-level or aggregated data should dominate.

Some general issues need to be addressed, limitations regarding sampling design, interviewer bias, definitions of socio-demographic factors and ceiling effect have been discussed elsewhere [8, 9]. Despite the above, there are limitations of modelling specification. As data were negatively skewed, the assumption of normality does not hold; though the estimates of parameters will still be consistent, the standard-error estimates will be inconsistent in small samples [50]. As there is correlation amongst the main effect dummy variables, models might suffer from multi-collinearity, heteroscedasticity and logically inconsistent in parameter estimates [16, 50, 51]. The potential extra health dimension might affect VAS values, and it might also be correlated with the EQ-5D dimensions, which can lead to bias in the estimations.

This study presents the feasibility of deriving an experience-based VAS values for EQ-5D-3L health states in China. The analysis of these VAS data raises more fundamental issues concerning the universal nature of the classification system and the extent to which Chinese respondents utilise the same concepts of health as defined by this classification system. Further investigation is needed regarding how the mode of administration, face-to-face interviews and the design of the VAS might influence responses. Additional analysis of these important population health survey data and qualitative studies may improve our understanding of these results but if, as seems probable, satisfactory explanations are not identified then more targeted studies of EQ-5D-3L focussing on these methodological issues would be justified.

## Electronic supplementary material

Below is the link to the electronic supplementary material.

Supplementary material 1 (PDF 84 kb)

## Abbreviations

QALY: Quality-adjusted life year
HRQoL: Health-related quality of life
NHSS: National Health Services Survey
SG: Standard gamble
RS: Rating scale
VAS: Visual analogue scale
TTO: Time trade-off
OLS: Ordinary least square
MAD: Mean absolute difference
SCC: Spearman rank correlation coefficients
