# Supplementary material for: Experience-based VAS values for EQ-5D-3L health states in a national general population health survey in China
Source: Qual Life Res. 2014 Sep 24;24(3):693–703. doi: 10.1007/s11136-014-0793-6 (PMC4349948; doi:10.1007/s11136-014-0793-6)

Online Resource

JOURNAL: Quality of Life Research (2014)

Experience-based VAS values for EQ-5D-3L health states in a national general population health survey in China

Authors:

Sun Sun [1, 2, 3]

Jiaying Chen [4]

Paul Kind [5]

Ling Xu [6]

Yaoguang Zhang [6]

Kristina Burström [1,2, 3]

[1] Department of Learning, Informatics, Management and Ethics, Medical Management Centre, Karolinska Institutet, Sweden

[2] Department of Public Health Sciences, Equity and Health Policy Research Group, Karolinska Institutet, Sweden

[3] Stockholm County Council, Health Care Services, Sweden

[4] School of Health Policy and Management, Nanjing Medical University, China

[5] Leeds Institute of Health Sciences, University of Leeds, UK

[6] Centre for Health Statistics and Information, Ministry of Health, China

Corresponding author:

Sun Sun

Karolinska Institutet

Department of Learning, Informatics, Management and Ethics

Tomtebodavägen 18A, SE-171 77 Stockholm, Sweden

Tel: +46 8 123 372 76

Email: sun.sun@ki.se

Supplementary Table S1 Observed health states (number of observations in each health state ≥ 5)

Profile	n	Mean	SD	Min	Max	Profile	n	Mean	SD	Min	Max
**11111**	105,033	82.6	11.9	0	100	**12122**	24	59.0	15.7	20	85
**11121**	3,789	69.1	13.3	0	100	**11311**	23	65.9	17.2	40	90
**11112**	2,220	70.8	14.4	0	100	**33321**	22	38.9	23.3	0	85
**11122**	1,676	63.0	14.0	6	100	**21321**	21	52.9	13.8	30	90
**22222**	1,275	54.7	14.5	4	100	**12212**	19	56.8	13.8	30	80
**21111**	676	69.6	14.3	1	100	**23332**	19	47.4	14.5	20	80
**22221**	599	60.2	13.4	20	90	**23311**	17	49.4	24.1	0	100
**21121**	567	65.1	13.3	0	100	**21232**	15	50.7	10.3	30	70
**21221**	403	60.2	13.9	20	99	**22332**	15	39.7	15.6	10	60
**21222**	345	55.1	13.9	10	85	**21311**	14	65.0	17.0	30	100
**11222**	316	58.3	14.0	5	95	**33311**	14	58.6	26.5	0	90
**22211**	299	63.7	15.4	3	100	**12112**	13	63.1	16.0	40	90
**11221**	296	62.4	14.1	30	90	**22312**	13	49.6	21.1	10	80
**11211**	294	69.2	12.7	15	100	**23221**	13	52.7	15.4	30	80
**21122**	270	59.4	14.3	0	90	**22112**	12	59.6	12.5	40	80
**21211**	215	64.8	13.5	10	100	**22323**	12	34.2	14.4	10	60
**22322**	129	48.3	16.4	10	80	**23222**	12	47.5	12.2	30	70
**12222**	111	59.0	15.0	20	90	**32333**	12	31.7	17.0	10	60
**11113**	99	63.0	19.2	10	100	**11321**	11	59.5	9.6	40	70
**33333**	91	34.0	17.9	0	80	**21223**	11	44.5	13.7	30	70
**22212**	88	57.3	13.9	30	90	**32321**	11	53.6	21.1	20	80
**12221**	87	63.3	11.8	30	90	**11133**	10	49.0	19.1	10	70
**33322**	82	39.9	18.5	0	80	**21132**	10	55.0	16.5	20	80
**11212**	76	61.6	15.1	10	90	**22333**	10	40.0	18.3	10	70
**12211**	76	67.1	12.3	30	100	**32221**	10	58.0	19.9	30	90
**12111**	74	73.8	15.2	20	100	**33323**	10	32.0	11.4	10	50
**22121**	62	62.4	14.0	30	90	**33331**	10	40.0	16.3	10	60
**21112**	57	60.8	16.9	0	90	**21231**	9	47.8	10.9	30	60
**21212**	57	58.1	13.3	20	85	**22231**	9	48.9	19.0	10	70
**23322**	56	44.6	15.1	10	75	**11322**	8	53.1	9.6	40	70
**21322**	50	48.7	13.4	20	70	**12223**	8	51.3	11.3	30	60
**33332**	46	33.4	15.5	10	86	**33312**	8	45.0	17.7	20	70
**11123**	45	49.3	18.4	0	80	**11232**	7	62.9	11.1	40	70
**22111**	45	67.5	16.6	3	90	**13322**	7	52.9	12.5	30	60
**22122**	45	60.1	14.6	30	95	**23312**	7	52.9	17.0	30	70
**22321**	45	50.7	17.2	10	80	**32332**	7	35.7	11.3	20	50
**11131**	44	62.3	16.3	30	90	**13111**	6	78.3	21.4	40	100
**22223**	39	38.8	15.6	3	80	**21123**	6	43.3	12.1	20	50
**22232**	37	48.1	12.4	30	70	**21131**	6	64.2	9.2	50	75
**32322**	37	47.3	15.6	10	80	**23323**	6	35.0	10.5	20	50
**22311**	33	58.0	12.7	30	90	**32311**	6	60.8	18.0	30	80
**12121**	32	68.8	12.7	40	90	**11233**	5	44.0	8.9	30	50
**32222**	31	54.7	13.7	20	80	**12322**	5	62.0	19.2	40	90
**11132**	29	54.0	18.6	20	90	**21312**	5	56.0	18.2	30	80
**22233**	26	41.5	15.2	10	70	**22213**	5	56.0	11.4	40	70
**23333**	26	37.3	20.7	0	80	**33222**	5	62.0	11.0	50	70
**23321**	25	41.2	15.6	20	70						

Supplementary Table S2 Estimated and rescaled VAS values for EQ-5D health states

Based on Model 3 (individual-level data)								
**EQ-5D**	**Estimated value**	**Rescaled value**	**EQ-5D**	**Estimated value**	**Rescaled value**	**EQ-5D**	**Estimated value**	**Rescaled value**
**11111**	82.39	1.00	**21111**	76.00	0.92	**31111**	74.26	0.90
**11112**	74.00	0.89	**21112**	67.61	0.81	**31112**	65.86	0.79
**11113**	65.76	0.79	**21113**	59.37	0.70	**31113**	57.63	0.68
**11121**	71.33	0.86	**21121**	64.94	0.78	**31121**	63.19	0.75
**11122**	62.93	0.75	**21122**	56.54	0.67	**31122**	54.80	0.65
**11123**	54.70	0.64	**21123**	48.31	0.56	**31123**	46.56	0.54
**11131**	68.41	0.82	**21131**	62.02	0.74	**31131**	60.28	0.72
**11132**	60.02	0.71	**21132**	53.63	0.63	**31132**	51.88	0.61
**11133**	51.78	0.61	**21133**	45.39	0.52	**31133**	43.65	0.50
**11211**	76.50	0.92	**21211**	70.11	0.84	**31211**	68.37	0.82
**11212**	68.11	0.82	**21212**	61.72	0.73	**31212**	59.98	0.71
**11213**	59.87	0.71	**21213**	53.48	0.63	**31213**	51.74	0.61
**11221**	65.44	0.78	**21221**	59.05	0.70	**31221**	57.31	0.68
**11222**	57.04	0.67	**21222**	50.65	0.59	**31222**	48.91	0.57
**11223**	48.81	0.57	**21223**	42.42	0.49	**31223**	40.67	0.46
**11231**	62.52	0.74	**21231**	56.13	0.66	**31231**	54.39	0.64
**11232**	54.13	0.64	**21232**	47.74	0.56	**31232**	46.00	0.53
**11233**	45.89	0.53	**21233**	39.50	0.45	**31233**	37.76	0.43
**11311**	71.18	0.86	**21311**	64.79	0.77	**31311**	63.04	0.75
**11312**	62.78	0.75	**21312**	56.39	0.67	**31312**	54.65	0.64
**11313**	54.55	0.64	**21313**	48.16	0.56	**31313**	46.41	0.54
**11321**	60.11	0.71	**21321**	53.72	0.63	**31321**	51.98	0.61
**11322**	51.72	0.61	**21322**	45.33	0.52	**31322**	43.58	0.50
**11323**	43.48	0.50	**21323**	37.09	0.42	**31323**	35.35	0.40
**11331**	57.20	0.68	**21331**	50.81	0.59	**31331**	49.06	0.57
**11332**	48.80	0.57	**21332**	42.41	0.49	**31332**	40.67	0.46
**11333**	40.57	0.46	**21333**	34.18	0.38	**31333**	32.43	0.36
**12111**	82.39	1.00	**22111**	76.00	0.92	**32111**	74.26	0.90
**12112**	74.00	0.89	**22112**	67.61	0.81	**32112**	65.86	0.79
**12113**	65.76	0.79	**22113**	59.37	0.70	**32113**	57.63	0.68
**12121**	71.33	0.86	**22121**	64.94	0.78	**32121**	63.19	0.75
**12122**	62.93	0.75	**22122**	56.54	0.67	**32122**	54.80	0.65
**12123**	54.70	0.64	**22123**	48.31	0.56	**32123**	46.56	0.54
**12131**	68.41	0.82	**22131**	62.02	0.74	**32131**	60.28	0.72
**12132**	60.02	0.71	**22132**	53.63	0.63	**32132**	51.88	0.61
**12133**	51.78	0.61	**22133**	45.39	0.52	**32133**	43.65	0.50
**12211**	76.50	0.92	**22211**	70.11	0.84	**32211**	68.37	0.82
**12212**	68.11	0.82	**22212**	61.72	0.73	**32212**	59.98	0.71
**12213**	59.87	0.71	**22213**	53.48	0.63	**32213**	51.74	0.61
**12221**	65.44	0.78	**22221**	59.05	0.70	**32221**	57.31	0.68
**12222**	57.04	0.67	**22222**	50.65	0.59	**32222**	48.91	0.57
**12223**	48.81	0.57	**22223**	42.42	0.49	**32223**	40.67	0.46
**12231**	62.52	0.74	**22231**	56.13	0.66	**32231**	54.39	0.64
**12232**	54.13	0.64	**22232**	47.74	0.56	**32232**	46.00	0.53
**12233**	45.89	0.53	**22233**	39.50	0.45	**32233**	37.76	0.43
**12311**	71.18	0.86	**22311**	64.79	0.77	**32311**	63.04	0.75
**12312**	62.78	0.75	**22312**	56.39	0.67	**32312**	54.65	0.64
**12313**	54.55	0.64	**22313**	48.16	0.56	**32313**	46.41	0.54
**12321**	60.11	0.71	**22321**	53.72	0.63	**32321**	51.98	0.61
**12322**	51.72	0.61	**22322**	45.33	0.52	**32322**	43.58	0.50
**12323**	43.48	0.50	**22323**	37.09	0.42	**32323**	35.35	0.40
**12331**	57.20	0.68	**22331**	50.81	0.59	**32331**	49.06	0.57
**12332**	48.80	0.57	**22332**	42.41	0.49	**32332**	40.67	0.46
**12333**	40.57	0.46	**22333**	34.18	0.38	**32333**	32.43	0.36
**13111**	79.18	0.96	**23111**	72.79	0.88	**33111**	71.05	0.85
**13112**	70.78	0.85	**23112**	64.39	0.77	**33112**	62.65	0.75
**13113**	62.55	0.75	**23113**	56.16	0.66	**33113**	54.42	0.64
**13121**	68.11	0.82	**23121**	61.72	0.73	**33121**	59.98	0.71
**13122**	59.72	0.71	**23122**	53.33	0.63	**33122**	51.59	0.60
**13123**	51.48	0.60	**23123**	45.09	0.52	**33123**	43.35	0.50
**13131**	65.20	0.78	**23131**	58.81	0.70	**33131**	57.07	0.67
**13132**	56.80	0.67	**23132**	50.41	0.59	**33132**	48.67	0.57
**13133**	48.57	0.57	**23133**	42.18	0.48	**33133**	40.44	0.46
**13211**	73.29	0.88	**23211**	66.90	0.80	**33211**	65.16	0.78
**13212**	64.90	0.78	**23212**	58.51	0.69	**33212**	56.76	0.67
**13213**	56.66	0.67	**23213**	50.27	0.59	**33213**	48.53	0.57
**13221**	62.23	0.74	**23221**	55.84	0.66	**33221**	54.09	0.64
**13222**	53.83	0.63	**23222**	47.44	0.55	**33222**	45.70	0.53
**13223**	45.59	0.53	**23223**	39.21	0.45	**33223**	37.46	0.42
**13231**	59.31	0.70	**23231**	52.92	0.62	**33231**	51.18	0.60
**13232**	50.92	0.60	**23232**	44.53	0.51	**33232**	42.78	0.49
**13233**	42.68	0.49	**23233**	36.29	0.41	**33233**	34.55	0.39
**13311**	67.96	0.81	**23311**	61.57	0.73	**33311**	59.83	0.71
**13312**	59.57	0.71	**23312**	53.18	0.62	**33312**	51.44	0.60
**13313**	51.33	0.60	**23313**	44.94	0.52	**33313**	43.20	0.50
**13321**	56.90	0.67	**23321**	50.51	0.59	**33321**	48.77	0.57
**13322**	48.50	0.56	**23322**	42.11	0.48	**33322**	40.37	0.46
**13323**	40.27	0.46	**23323**	33.88	0.38	**33323**	32.14	0.35
**13331**	53.98	0.64	**23331**	47.60	0.55	**33331**	45.85	0.53
**13332**	45.59	0.53	**23332**	39.20	0.45	**33332**	37.46	0.42
**13333**	37.35	0.42	**23333**	30.96	0.34	**33333**	29.22	0.32

to be continued

continued

Based on Model 1M1 (aggregated data)								
**EQ-5D**	**Estimated value**	**Rescaled value**	**EQ-5D**	**Estimated value**	**Rescaled value**	**EQ-5D**	**Estimated value**	**Rescaled value**
**11111**	74.12	1.00	**21111**	69.63	0.94	**31111**	68.24	0.92
**11112**	69.00	0.93	**21112**	64.51	0.86	**31112**	63.12	0.84
**11113**	61.73	0.82	**21113**	57.24	0.76	**31113**	55.85	0.74
**11121**	67.77	0.91	**21121**	63.28	0.84	**31121**	61.89	0.82
**11122**	62.64	0.84	**21122**	58.15	0.77	**31122**	56.76	0.75
**11123**	55.38	0.73	**21123**	50.89	0.67	**31123**	49.49	0.65
**11131**	64.28	0.86	**21131**	59.79	0.79	**31131**	58.39	0.77
**11132**	59.15	0.78	**21132**	54.66	0.72	**31132**	53.27	0.70
**11133**	51.88	0.68	**21133**	47.39	0.62	**31133**	46.00	0.60
**11211**	69.27	0.93	**21211**	64.78	0.87	**31211**	63.39	0.85
**11212**	64.15	0.86	**21212**	59.66	0.79	**31212**	58.26	0.77
**11213**	56.88	0.75	**21213**	52.39	0.69	**31213**	51.00	0.67
**11221**	62.92	0.84	**21221**	58.43	0.77	**31221**	57.04	0.75
**11222**	57.79	0.77	**21222**	53.30	0.70	**31222**	51.91	0.68
**11223**	50.52	0.66	**21223**	46.03	0.60	**31223**	44.64	0.58
**11231**	59.42	0.79	**21231**	54.93	0.72	**31231**	53.54	0.70
**11232**	54.30	0.72	**21232**	49.81	0.65	**31232**	48.42	0.63
**11233**	47.03	0.61	**21233**	42.54	0.55	**31233**	41.15	0.53
**11311**	64.33	0.86	**21311**	59.84	0.79	**31311**	58.45	0.77
**11312**	59.21	0.79	**21312**	54.72	0.72	**31312**	53.32	0.70
**11313**	51.94	0.68	**21313**	47.45	0.62	**31313**	46.06	0.60
**11321**	57.98	0.77	**21321**	53.49	0.70	**31321**	52.10	0.68
**11322**	52.85	0.69	**21322**	48.36	0.63	**31322**	46.97	0.61
**11323**	45.59	0.59	**21323**	41.10	0.53	**31323**	39.70	0.51
**11331**	54.49	0.72	**21331**	50.00	0.65	**31331**	48.60	0.63
**11332**	49.36	0.64	**21332**	44.87	0.58	**31332**	43.48	0.56
**11333**	42.09	0.54	**21333**	37.60	0.48	**31333**	36.21	0.46
**12111**	74.91	1.01	**22111**	70.42	0.95	**32111**	69.02	0.93
**12112**	69.78	0.94	**22112**	65.29	0.87	**32112**	63.90	0.85
**12113**	62.51	0.83	**22113**	58.02	0.77	**32113**	56.63	0.75
**12121**	68.55	0.92	**22121**	64.06	0.86	**32121**	62.67	0.84
**12122**	63.42	0.85	**22122**	58.93	0.78	**32122**	57.54	0.76
**12123**	56.16	0.74	**22123**	51.67	0.68	**32123**	50.28	0.66
**12131**	65.06	0.87	**22131**	60.57	0.81	**32131**	59.18	0.79
**12132**	59.93	0.80	**22132**	55.44	0.73	**32132**	54.05	0.71
**12133**	52.66	0.69	**22133**	48.17	0.63	**32133**	46.78	0.61
**12211**	70.05	0.94	**22211**	65.56	0.88	**32211**	64.17	0.86
**12212**	64.93	0.87	**22212**	60.44	0.80	**32212**	59.05	0.78
**12213**	57.66	0.76	**22213**	53.17	0.70	**32213**	51.78	0.68
**12221**	63.70	0.85	**22221**	59.21	0.79	**32221**	57.82	0.77
**12222**	58.57	0.78	**22222**	54.08	0.71	**32222**	52.69	0.69
**12223**	51.31	0.67	**22223**	46.82	0.61	**32223**	45.42	0.59
**12231**	60.21	0.80	**22231**	55.72	0.74	**32231**	54.32	0.72
**12232**	55.08	0.73	**22232**	50.59	0.66	**32232**	49.20	0.64
**12233**	47.81	0.62	**22233**	43.32	0.56	**32233**	41.93	0.54
**12311**	65.11	0.87	**22311**	60.62	0.81	**32311**	59.23	0.79
**12312**	59.99	0.80	**22312**	55.50	0.73	**32312**	54.11	0.71
**12313**	52.72	0.69	**22313**	48.23	0.63	**32313**	46.84	0.61
**12321**	58.76	0.78	**22321**	54.27	0.71	**32321**	52.88	0.69
**12322**	53.63	0.71	**22322**	49.14	0.64	**32322**	47.75	0.62
**12323**	46.37	0.60	**22323**	41.88	0.54	**32323**	40.49	0.52
**12331**	55.27	0.73	**22331**	50.78	0.66	**32331**	49.38	0.64
**12332**	50.14	0.66	**22332**	45.65	0.59	**32332**	44.26	0.57
**12333**	42.87	0.55	**22333**	38.38	0.49	**32333**	36.99	0.47
**13111**	67.14	0.90	**23111**	62.65	0.84	**33111**	61.26	0.82
**13112**	62.01	0.83	**23112**	57.52	0.76	**33112**	56.13	0.74
**13113**	54.74	0.72	**23113**	50.25	0.66	**33113**	48.86	0.64
**13121**	60.78	0.81	**23121**	56.29	0.74	**33121**	54.90	0.72
**13122**	55.66	0.73	**23122**	51.17	0.67	**33122**	49.77	0.65
**13123**	48.39	0.63	**23123**	43.90	0.57	**33123**	42.51	0.55
**13131**	57.29	0.76	**23131**	52.80	0.69	**33131**	51.41	0.67
**13132**	52.16	0.68	**23132**	47.67	0.62	**33132**	46.28	0.60
**13133**	44.90	0.58	**23133**	40.41	0.52	**33133**	39.01	0.50
**13211**	62.29	0.83	**23211**	57.80	0.77	**33211**	56.41	0.75
**13212**	57.16	0.76	**23212**	52.67	0.69	**33212**	51.28	0.67
**13213**	49.89	0.65	**23213**	45.40	0.59	**33213**	44.01	0.57
**13221**	55.93	0.74	**23221**	51.44	0.67	**33221**	50.05	0.65
**13222**	50.80	0.67	**23222**	46.31	0.60	**33222**	44.92	0.58
**13223**	43.54	0.56	**23223**	39.05	0.50	**33223**	37.66	0.48
**13231**	52.44	0.69	**23231**	47.95	0.62	**33231**	46.56	0.60
**13232**	47.31	0.61	**23232**	42.82	0.55	**33232**	41.43	0.53
**13233**	40.05	0.51	**23233**	35.56	0.45	**33233**	34.16	0.43
**13311**	57.35	0.76	**23311**	52.86	0.69	**33311**	51.47	0.67
**13312**	52.22	0.69	**23312**	47.73	0.62	**33312**	46.34	0.60
**13313**	44.95	0.58	**23313**	40.46	0.52	**33313**	39.07	0.50
**13321**	50.99	0.67	**23321**	46.50	0.60	**33321**	45.11	0.58
**13322**	45.86	0.59	**23322**	41.37	0.53	**33322**	39.98	0.51
**13323**	38.60	0.49	**23323**	34.11	0.43	**33323**	32.72	0.41
**13331**	47.50	0.62	**23331**	43.01	0.55	**33331**	41.62	0.53
**13332**	42.37	0.54	**23332**	37.88	0.48	**33332**	36.49	0.46
**13333**	35.11	0.44	**23333**	30.62	0.38	**33333**	29.22	0.36

Split sample test

A split sample test was used to estimate the robustness of the final model. The total sample was randomly divided into two groups of equal size [16]. The estimations from one group were then used to predict values for health states in the other group.

The results from the split sample test are presented in Table S2. For health states with five or more observations, for models based on individual-level data, the SCC was 0.901 and MAD was 4.44. For models based on aggregated data, the SCC was 0.855 and MAD was 4.11. For both types of models, the results from the split sample test showed a smaller SCC and greater MAD. For health states with ten or more observations, for models based on individual-level data, the results from the split sample test were similar to that of the whole sample; for models based on aggregated data, the results for split sample test showed a smaller SCC and greater MAD.

Supplementary Table S3 Spearman rank correlation coefficients (SCC) and mean absolute difference (MAD), for whole sample and split sample, respectively

	n≥5				n≥10			
	Whole Sample		Split sample		Whole Sample		Split sample	
	SCC	MAD	SCC	MAD	SCC	MAD	SCC	MAD
**Model 3**	0.913	4.18	0.901	4.44	0.939	4.08	0.943	4.03
**Model 1M5**	0.928	3.10	0.855	4.11	0.951	2.64	0.923	3.42

Effect of socio-demographic factors on health state valuation

By including age, sex, region, marital status, educational level, occupational status and income groups in the final model based on the individual-level data (Model 3), we investigated the effect of socio-demographic factors on health state valuation and the robustness of the model (Table S4). The classification of socio-demographic variables is described elsewhere [8, 9].

Supplementary Table S4 Effect of age, sex, region and socio-economic characteristics on health state values (all results were statistically significant [p < 0.05] except those shaded)

	Model 3A	Model 3B	Model 3C	Model 3D	Model 3E	Model 3F	Model 3G	Model 3H	Model 3I
**Intercept**	89.39	90.04	89.40	90.18	87.67	89.75	88.46	88.04	86.54
**Mobility**									
Level 2	-4.25	-4.27	-4.19	-4.25	-4.27	-4.20	-4.18	-4.17	-4.10
Level 3	-6.92	-6.97	-7.05	-6.98	-7.08	-6.97	-6.93	-7.11	-7.13
**Self-care**									
Level 3*	-2.62	-2.64	-2.72	-2.62	-2.78	-2.56	-2.82	-2.67	-2.73
**Usual activities**									
Level 2	-4.49	-4.51	-4.49	-4.50	-4.38	-4.40	-4.38	-4.31	-4.29
Level 3	-9.84	-9.94	-9.99	-9.96	-9.73	-9.69	-9.77	-9.53	-9.58
**Pain/discomfort**									
Level 2	-8.58	-8.47	-8.48	-8.46	-8.35	-8.47	-8.37	-8.35	-8.37
Level 3	-12.09	-11.91	-11.93	-11.93	-11.81	-11.90	-11.99	-11.77	-11.81
**Anxiety/depression**									
Level 2	-8.07	-8.03	-7.69	-8.00	-7.86	-7.99	-7.75	-7.81	-7.49
Level 3	-16.75	-16.75	-16.20	-16.71	-16.56	-16.64	-16.30	-16.49	-15.97
**Age group** [a]									
25-34	-2.70	-2.67	-2.63	-2.18	-2.48	-2.27	-2.76	-1.69	-1.65
35-44	-5.33	-5.31	-5.36	-4.73	-4.92	-4.91	-5.42	-4.00	-4.06
45-54	-8.54	-8.55	-8.68	-7.95	-7.98	-8.04	-8.73	-6.84	-7.04
55-64	-11.42	-11.46	-11.55	-10.83	-10.55	-10.80	-11.54	-9.03	-9.30
65-74	-14.61	-14.66	-14.75	-13.91	-13.53	-13.64	-14.75	-11.42	-11.81
75-103	-15.80	-15.81	-16.10	-14.88	-14.44	-14.55	-15.99	-11.89	-12.53
**Sex** [b]	–	-1.27	-1.30	-1.20	-0.98	-1.13	-1.30	-0.70	-0.76
**Region**									
Rural [c]	–	–	0.87	–	–	–	–	–	1.05
Middle [d]	–	–	3.06	–	–	–	–	–	2.87
Western	–	–	1.07	–	–	–	–	–	0.97
**Marital status** [e]									
Married	–	–	–	-0.73	–	–	–	-0.76	-0.72
Divorced	–	–	–	-2.37	–	–	–	-2.25	-1.83
Widowed	–	–	–	-1.54	–	–	–	-1.10	-1.01
Other	–	–	–	-2.24	–	–	–	-2.07	-1.64
**Educational level** [f]									
Primary school	–	–	–	–	1.05	–	–	1.21	1.10
Junior middle school	–	–	–	–	2.38	–	–	2.81	2.52
Senior middle school	–	–	–	–	2.33	–	–	2.91	2.74
College and above	–	–	–	–	2.34	–	–	2.94	2.93
Missing	–	–	–	–	1.93	–	–	3.11	2.89
**Occupational status** [g]									
Retired	–	–	–	–	–	-0.91	–	-1.99	-1.56
Student	–	–	–	–	–	1.00	–	0.62	0.57
Unemployed	–	–	–	–	–	-1.75	–	-1.82	-1.71
Missing	–	–	–	–	–	-4.36	–	-4.10	-3.83
**Income groups** [h]									
Second group	–	–	–	–	–	–	1.46	-0.36	-0.35
Third group	–	–	–	–	–	–	1.76	-0.61	-0.73
Fourth group	–	–	–	–	–	–	2.79	-1.95	-1.99
Fifth group (high)	–	–	–	–	–	–	2.71	-0.03	-0.35
**Observations**	120,709	120,709	120,709	120,709	120,709	120,709	120,709	120,709	120,709
**Adjusted R^2**	0.3401	0.3422	0.3494	0.3427	0.3453	0.3443	0.3475	0.3489	0.3568

* Reference group: self-care on level 1 or 2

[a] Reference group: age group 18-24 years

[b] Reference group: men

[c] Reference group: urban

[d] Reference group: eastern area

[e] Reference group: single

[f] Reference group: below primary school

[g] Reference group: employed

[h] Reference group: first group (low)

Supplementary Figure S5 Distribution of VAS values

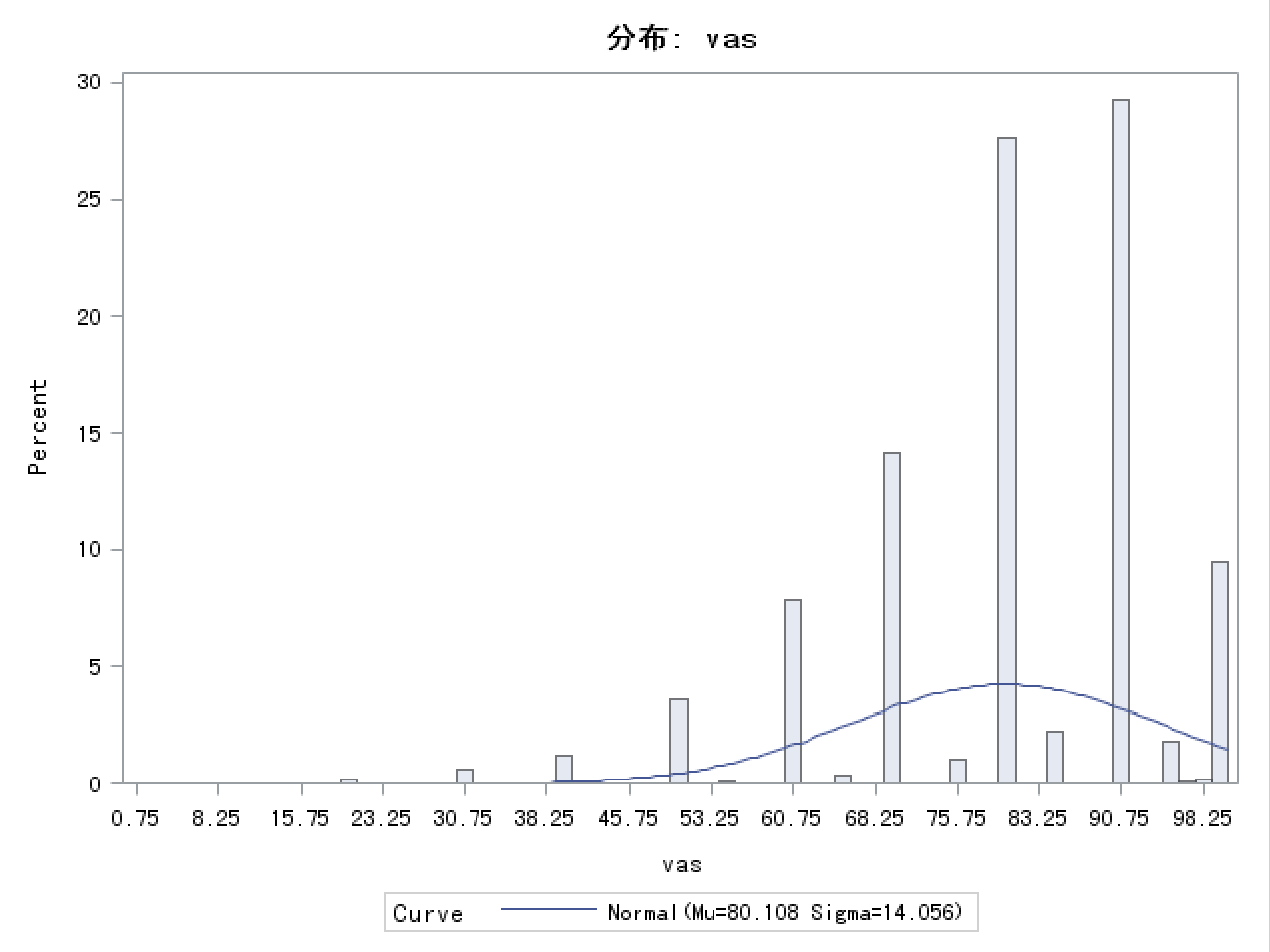

Supplementary Figure S6 Distribution of VAS values after log transformation

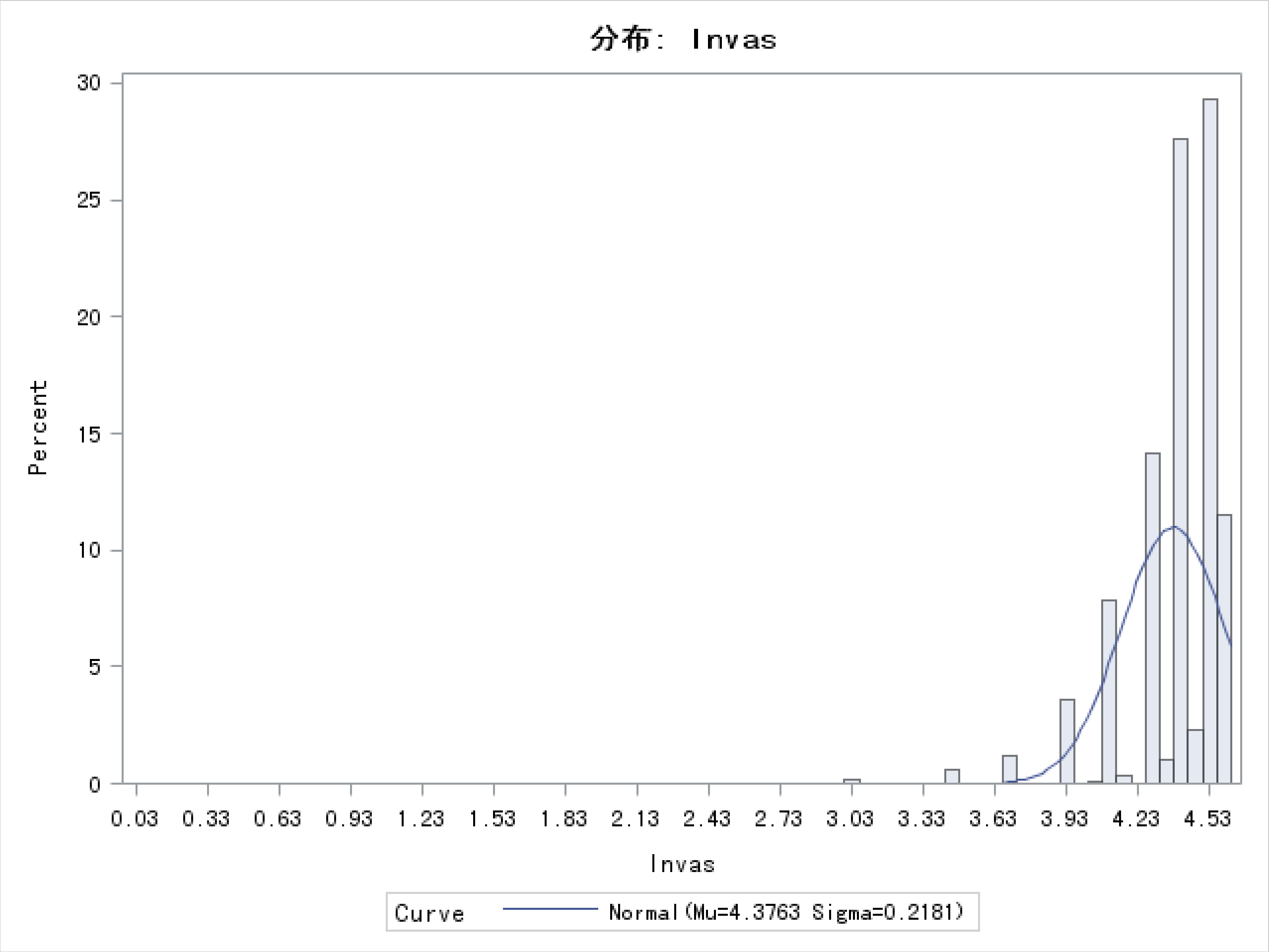

Supplement: Supplementary file 1 — Supplementary material 1 (PDF 84 kb) [file 11136_2014_793_MOESM1_ESM.pdf]
